# Linkage Analysis in Familial Non-Lynch Syndrome Colorectal Cancer Families from Sweden

**DOI:** 10.1371/journal.pone.0083936

**Published:** 2013-12-11

**Authors:** Vinaykumar Kontham, Susanna von Holst, Annika Lindblom

**Affiliations:** Department of Molecular Medicine and Surgery, Karolinska Institutet, Stockholm, Sweden; University of Illinois at Chicago, United States of America

## Abstract

Family history is a major risk factor for colorectal cancer and many families segregate the disease as a seemingly monogenic trait. A minority of familial colorectal cancer could be explained by known monogenic genes and genetic loci. Familial polyposis and Lynch syndrome are two syndromes where the predisposing genes are known but numerous families have been tested without finding the predisposing gene. We performed a genome wide linkage analysis in 121 colorectal families with an increased risk of colorectal cancer. The families were ascertained from the department of clinical genetics at the Karolinska University Hospital in Stockholm, Sweden and were considered negative for Familial Polyposis and Lynch syndrome. In total 600 subjects were genotyped using single nucleotide polymorphism array chips. Parametric- and non-parametric linkage analyses were computed using MERLIN in all and subsets of families. No statistically significant result was seen, however, there were suggestive positive HLODs above two in parametric linkage analysis. This was observed in a recessive model for high-risk families, at locus 9q31.1 (HLOD=2.2, rs1338121) and for moderate-risk families, at locus Xp22.33 (LOD=2.2 and HLOD=2.5, rs2306737). Using families with early-onset, recessive analysis suggested one locus on 4p16.3 (LOD=2.2, rs920683) and one on 17p13.2 (LOD/HLOD=2.0, rs884250). No NPL score above two was seen for any of the families. Our linkage study provided additional support for the previously suggested region on chromosome 9 and suggested additional loci to be involved in colorectal cancer risk. Sequencing of genes in the regions will be done in future studies.

## Introduction

Colorectal cancer (CRC) is increasing in incidence and is ranked as the second and third most common cancer type in the western world and Sweden respectively. CRC has a lifetime risk of 5% and affects men and women equally. One major risk factor is a family history of the disease and 20-25% of all CRC cases have a close relative with the same disease [1]. Known syndromes such as familial adenomatous polyposis (FAP) and Lynch syndrome are responsible for less than 5 % of colorectal cancer cases [2], which leaves a majority of the familial colorectal cancer cases unexplained. The inheritance often suggests a dominant transmission of the disease, but recessive inheritance and even a complex inheritance have been suggested [3]. A syndrome, familial CRC type X, has been suggested for families fulfilling criteria for Lynch syndrome but without germline mutations [4]. However, families negative after Lynch syndrome diagnostics only rarely fulfil these strict criteria, mostly because a later onset or reduced penetrance. Recently Genome Wide Association Studies (GWAS) have been used to find genetic loci associated with a certain risk to develop CRC. These loci; 6p21, 8q23.3, 8q24.21, 9p24, 10p14, 11q13.4, 11q23.1, 14q22.2, 15q13.3, 16q22.1, 18q21.1, 19q13.1 and 20p12.3, 1q41, 3q26.2, 12q13.13, 20q13.33, Xp22.2 [5-14] could to some degree support the explanation of CRC as a complex disease. Historically, linkage analysis has been a successful tool finding monogenic disease causing colorectal cancer genes like APC [15], MSH2 [16] and MLH1 [17]. Also new candidate regions for additional existence of moderate to high- penetrant CRC loci have been reported from linkage studies, but no casual mutation has yet been found. The loci on chromosome 9q [18-20], 3q [21,22] and 14q [23,24] have been reported more than once. A previous linkage study in familial serrated neoplasia suggested a locus on chromosome 2q [25]. Recently, 4 different loci with a significant HLOD above 3; on chromosomes 12q24 in all CRC families, 4q21 in early onset-, 15q22.31 in high-risk- and 8q13.2 in moderate-risk families was suggested [26]. The current study performed a genome wide linkage (GWL) scan using 600 individuals from 121 Swedish CRC families and analysed all families, early onset-, high-risk and moderate risk families separately. 

## Materials and Methods

### Ethics statement

The study was undertaken in agreement with the Swedish legislation of ethical permission and according to the decision in the Stockholm regional ethical committee (2008/125-31.2). All participants gave written informed consent to participate in the study.

### Patients

The families were ascertained through the clinical genetics department at the Karolinska University Hospital in Stockholm, Sweden between 1990 and 2005. FAP was excluded using medical records from affected individuals and Lynch syndrome was excluded using our current clinical protocol [27]. Families were included in the study if there was at least two affected relatives informative for linkage analysis, thus at least a sib-pair. Details on the families are shown in table 1. Early onset families were defined as families with a mean-age of diagnosis less than 50, high-risk families were defined as families with three or more affected individuals in close relatives. Moderate risk families were defined as families with two or more sibs affected. Eight families fulfilled criteria for CRC type X [4] (two overlapped with early onset families) but were not analysed separately.

**Table 1:** Description of families in linkage analysis.

	Total	>3 CRC	mean <50	Sibs only
**No. families**	121	27	8	49
**Mean age**	64	62	48	66
**No. families with any <50**	27	8	8	6
**No. families with any <60**	72	21	8	22

### Genotyping

Genomic DNA was extracted from peripheral blood using standard procedures. Genotyping was done separately in two different sets of family material. Genotyping of 548 patients with 6090 markers was performed by the Illumina Infinium assay [28,29] using the Illumina HumanLinkage-12 DNA analysis bead chip. The overall reproducibility in the genotype data was 99.996% based on 6.25% of duplicated genotypings. The average call rate per SNP was 99.57%. Additionally, 52 subjects were genotyped using the Illumina Golden Gate assay [30] and the Illumina Linkage Panel IVb (6008 markers). The overall reproducibility in the genotype data was 100% based on 2.2% of duplicated genotypings. The average call rate per SNP was 97.27%. Arrays were processed according to manufactures protocol at the SNP Technology Platform in Uppsala and available upon request. 

### Linkage analysis

Pedcheck [31] was used to check for the initial Mendelian inheritance analysis among the families. The family based genetic model was used for parametric linkage analysis for all chromosomes, including chromosome X. As a supplement non-parametric analysis using Whittemore and Halpern NPL statistics was made [32]. LOD scores as well as heterogeneity LOD scores were computed using MERLIN (version 1.1.2) [33] and was given for all genotyped positions. Analyses were done assuming both dominant and recessive traits. For autosomal dominant and recessive mode of inheritance the disease allele frequency was set to 0.0001. The penetrance rates for the dominant and recessive mode of inheritance for homozygous normal, heterozygous, and homozygous affected were set to 0.05, 0.80, 0.80 and 0.001, 0.001, 1.0 respectively.

Individuals with colorectal cancer or a polyp with high degree dysplasia were coded as affected. Family members with unclear status were coded as unknown. Families were ascertained assuming a dominant trait and spouses were therefore coded as unaffected. Four different analyses were performed using different sets of families and patients; all families, all families with at least three cases (high-risk), all families with CRC among sibs (moderate-risk) and families with a mean age below 50 (early-onset) to be comparable with the results from the recent linkage study by Cicek et al.

All 121 families, including all subjects from both genotyping sessions, were used for linkage analysis. Thus, two marker files were merged and 7256 markers were used in the analysis. Merlin by default allows a maximum of 24 bits for each family, why four large families had to be split. The families were split so that each sub-family used one common ancestor and fitted into the limit as defined while running the program. The original 121 families were analysed as 126 (one family had to be split into three). 

Since presence of linkage disequilibrium (LD) may inflate multipoint linkage statistics, a threshold of r^2^ = 0.1 has been used to avoid that false positive results inflate the statistics [34]. LD among SNPs with r^2^>0.1 was accounted for, by MERLIN organizing the markers into clusters. MERLIN makes use of the population haplotype frequencies to assume LD within each cluster. To maintain uniformity in our study subsets, the same clusters were continuously used in all analysis. 

## Results

**Figure 1:**
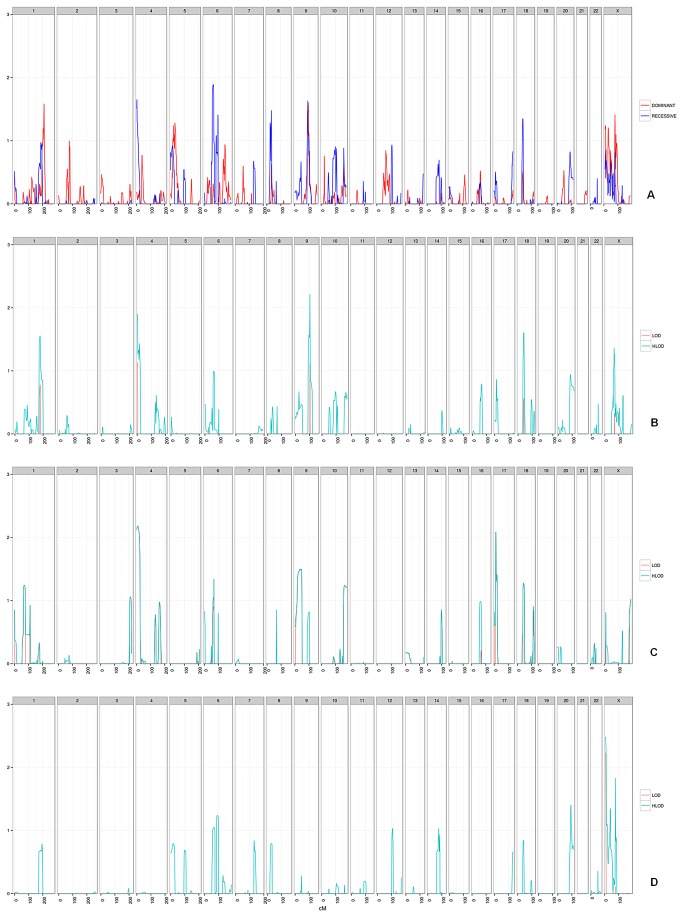
Plot of LOD / HLOD*. a) Plot of HLODs for all families in the study, dominant (in Red) and recessive (in Blue) models (n=121). b) LOD/HLOD plot of study group with more than 3 affected individuals (n=27). c) LOD/HLOD plot of study group with mean age of diagnosis < 50 (n=8). d) LOD/HLOD plot of study group with affected sibs (n=49). * -negative values of scores are not plotted shown. For b,c,d - LODs are represented in Red, HLODs are represented in Cyan.

A total of 600 individuals from 121 families were successfully genotyped. The analyses were conducted for all and each of three different subgroups; high-risk, moderate-risk and early-onset families (table 1). There was no individual statistically significant (above three) LOD score or HLOD in any of the analysis (Figure 1). However, there were positive HLODs above two (table 2). 

**Table 2:** Summary of Colorectal cancer linkage results with maximum observed HLODs greater than 2.

**Study Group**	No. Of Families	Linked Region	cM, SNP	Model	HLOD (α)
**All families**	121	-	-	-	-
**More than 3 affected**	27	9q31.1	102.68, RS1338121	Recessive	2.212 (0.66)
**Mean age at Diagnosis < 50**	8	4p16.3	7.17, RS920683	Recessive	2.184 (1.00)
		17p13.2	11.51, RS884250	Recessive	2.086 (1.00)
**Families with Affected Sibs**	49	Xp22.33	7.42, RS2306737	Recessive	2.486 (0.79)

For high-risk families (in total 27 families) one locus on chromosome 9q31.1, showed an HLOD above two assuming recessive inheritance and an estimated 66% of families linked (table 2). Maximum was for the marker rs1338121 with a HLOD of 2.2. LOD on the same locus in recessive model was 0.7. For dominant disease this locus showed a LOD score of 1.4 and an HLOD of 1.6. 

For moderate-risk families (in total 49 families, analysed as 50) one locus on the tip on chromosome Xp had LOD and HLOD above two with maximum 2.2 and 2.5 respectively, for the marker rs2306737 in recessive analysis (table 2). LOD score and HLOD for dominant analysis were both 1.8. 

Finally, for the group of families with early-onset (only 8 families) two loci showed positive LOD scores and HLODs above two in recessive analysis (table 2). One was distal on chromosome 4p with a maximum LOD and HLOD of 2.2 for rs920683 and the other was on chromosome 17p13.2 with a maximum LOD and HLOD of 2.0 for rs884250. The LOD and HLOD for dominant analysis was 0.976 on chromosome 4p and 1.25 on chromosome 17p13.2. The LODs were similar in parametric and nonparametric analysis and 100% linked families were assumed for both loci. 

No NPL score above two was seen for any of the families. HLODs above 2 are presented in table 2. All loci with an HLOD>1.0 in parametric analysis are shown in table 3. 

**Table 3:** Summary of Colorectal cancer linkage results with maximum observed HLODs between 1 and 2.

**Study Group**	No. Of Families	Linked Region	cM, SNP	Model	HLOD (α)
**All Families**	121	1q32.1	204.36, RS2032018	Dominant	1.191 (0.46)
		5p15.2	33.99, RS879253	Dominant	1.258 (0.48)
		9q22.31	97.96, RS4534181	Dominant	1.602 (0.59)
			94.37, RS7037744	Recessive	1.632 (0.32)
		Xp11.21	79.25, RS2015312	Dominant	1.414 (0.72)
		4p16.3	2.97, RS736455	Recessive	1.653 (0.38)
		6p21.1	64.36, RS722269	Recessive	1.892 (0.28)
		8p22	33.01, RS334206	Recessive	1.479 (0.26)
		18p11.21	40.13, RS1043925	Recessive	1.351 (0.27)
**More than 3 affected**	27	1q32.2	211.46, RS1507765	Dominant	1.414 (0.61)
		2p16.2	77.83, RS1483869	Dominant	1.438 (0.60)
		4q28.3	134.94, RS426029	Dominant	1.045 (0.54)
		5p15.1	34.80, RS1505034	Dominant	1.678 (0.71)
		9q31.1	102.68, RS1338121	Dominant	1.672 (0.79)
		12q13.12	63.98, RS7532	Dominant	1.086 (0.51)
		16q12.2	65.68, RS1990637	Dominant	1.556 (0.77)
		Xp11.21	79.24, RS1560514	Dominant	1.788 (1.00)
		1q25.2	178.47, RS227530	Recessive	1.554 (0.55)
		4p16.3	2.97, RS736455	Recessive	1.903 (0.68)
		18p11.21	40.13, RS1043925	Recessive	1.605 (0.48)
		Xp11.3	67.42, RS1137070	Recessive	1.267 (0.56)
**Mean age at Diagnosis < 50**	8	2p16.3	74.90, RS1394207	Dominant	1.145 (1.00)
		9p21.3	45.61, RS10757309	Dominant	1.614 (1.00)
		10q22.1	86.06, RS1227938	Dominant	1.200 (1.00)
		16q21	80.78, RS17822576	Dominant	1.181 (1.00)
		17p13.1	24.87, RS1391766	Dominant	1.258 (1.00)
		1p33	72.10, RS1934405	Recessive	1.244 (1.00)
		6p21.1	67.58, RS4714772	Recessive	1.335 (1.00)
		9p21.3	45.61, RS10757309	Recessive	1.501 (1.00)
		10q26.3	166.39, RS7072831	Recessive	1.239 (0.90)
		18q11.2	42.92, RS12959039	Recessive	1.279 (1.00)
**Families with Affected Sibs**	49	4p15.2	38.72, RS216113	Dominant	1.112 (0.96)
		6q23.3	136.59, RS975676	Dominant	1.848 (1.00)
		Xp22.33	11.69, RS749706	Dominant	1.860 (1.00)
		6q14.1	91.67, RS885582	Recessive	1.241 (0.33)
		12q23.1	107.90, RS17290272	Recessive	1.034 (0.26)
		14q24.3	78.12, RS888412	Recessive	1.032 (0.29)
		20q13.31	93.25, RS186659	Recessive	1.410 (0.37)

## Discussion

We used SNP genotyping to perform a linkage analysis in 121 CRC families and did not find any overall statistically significant results with a LOD or HLOD over 3. A few large families were split to be able to use MERLIN, and lost a bit of its power in the analysis. The effect of this was of little importance. 

However, we did find LODs and HLODs above 2, suggestive of linkage. A previous linkage study used 356 families and showed one locus with HLOD > 3 (12q) and 4 with HLOD > 2 (on chromosomes 4q, 15q, 17q and 12q), all in dominant analysis [26]. We found no support for any of these regions in our analysis. 

In our substudy of large, high-risk families (more than three affecteds), a locus on chromosome 9q31 was found. The same region has been suggested before, although in dominant models, and was here again identified by us using linkage analysis in a recessive model. This locus was previously suggested by one sib-pair study and one study using linkage in many kindred and also previously by us in one large family with rectal cancer and adenomas [18-20]. Those three studies used microsatellites for genotyping while previous linkage studies using SNPs were unable to replicate the locus [18,26,35]. One study has also shown support for the region using a five-SNP haplotype in the region [36]. Two genes have so far been suggested as the predisposing genes in this region. First, it was suggested that germline allele-specific expression resulted in reduced expression of the gene altering SMAD-mediated TGF-beta signaling [37]. Secondly, a study of the GALNT12 gene demonstrated truncating somatic and germline mutations in CRC patients but none in controls and genetic defects in the O-glycosylation pathway in part underlie aberrant glycosylation, and thereby contribute to the development in a subset of CRC [38]. The region suggested by our present study overlaps well with the region suggested by one family before [19]. The region spans over almost 9 Mb and includes the above mentioned genes and numerous of others. Four families contribute mostly to the positive, dominant and recessive HLOD score. Only one family had early-onset disease. The Cicek study defined for a similar group of 67 families one locus with HLOD >3 (15q) and 4 with HLOD > 2 (chromosomes 12q, 14q, 17q and Xp) some using dominant or recessive (chromosome 14) model. We found no support for any of those regions in our study. However, we had an HLOD>1 close to the chromosome X region. 

For moderate-risk families, with affected sibs only, one region on the tip on chromosome X was suggested in recessive model (max HLOD=2.2) and similar to the chromosome 9 locus there was also a positive but lower LOD using a dominant model (0.7). This region is almost 6 Mb, has not been suggested before and contains numerous candidate genes. The Cicek study included 200 moderate risk families and also using recessive model they identified one locus with an HLOD>3 (8q) and 4 loci with an HLOD>2 (chromosomes 1q, 6p, 8q and 22q). We could not find support for any of those loci. 

Finally we observed using a recessive model two loci with high HLODs (4p and 17p) for the early-onset families. However, this group consisted of only 8 families. Family 8 was also among the high-risk families linked to chromosome 9 locus and for this size of family it is expected that a whole genome study should generate linkage to many regions and thus most will be false positives. We could not replicate any of the loci from Cicek et al. using dominant or recessive model (chromosomes 4q, 14q, 15q and 22q).

A previous study focusing on adenoma and colorectal adenoma and carcinoma found linkage to chromosomal regions on chromosomes 18q21 and 2p22 in 69 families while a sub-analysis of 55 families with cancer only showed linkage to chromosome 3q21-24[21]. None of these regions were confirmed by the Cicek study or by this study. It is surprising that 4 linkage studies including this, all using SNP-markers do not generate any overlapping loci [21,26,35]. There are several differences between the studies though, which are possible explanations for this discrepancy. The ethnicity of the subjects in the studies is different, one study is from USA, one is from the Netherland’s, one from UK and our study is based on the Swedish population. The sample sizes are also different, the US study have in total 356 families, the Dutch only seven large families, the UK study 69 and our study 121 families. The recruitment process also differed between all four studies, but in general most of the different results could be explained also by biology and different predisposing elements in each sample sets. Future experiments need to consider this heterogeneity in their design.

Slightly to our surprise we could see also positive parametric LOD-scores in this study compared to our previous ones [22,23] where only HLODs were obtained. This might be related to the fact that we used SNPs instead of microsatellites in this study. Microsatellites are much more informative and thus often very low negative LODs are seen when a family is not linked. In this SNP oriented experiment few families had LODs below -2 and thus the power to exclude linkage in our experiment was low. However, power to detect linkage was retained (which we tested using our family 24 with a LOD of 3 for the chromosome 9 locus) [19]. The result could also relate to the fact that families in this study were smaller compared to our previous studies using 20 and 30 large pedigrees [22,23]. Here we used a different strategy with 121 smaller families and in contrast to our previous linkage studies we could find support to the candidate region on chromosome 9. We previously used SimWalk2 for linkage analysis with microsatellite markers but it was not conducive for analysis of SNP data. To justify using MERLIN, we analysed family 24 and chromosome 9 using SimWalk2 (data not shown), which took three weeks to complete but obtained identical results. 

In conclusion, our linkage study provided additional support for the region on chromosome 9, some evidence for new loci to be involved in colorectal cancer risk and no support for other previously reported loci. This suggested heterogeneity if familial CRC should influence the design of future association and linkage studies.
